# Impact of endocrine disrupting chemicals and pharmaceuticals on Sertoli cell development and functions

**DOI:** 10.3389/fendo.2023.1095894

**Published:** 2023-01-30

**Authors:** Maia Corpuz-Hilsabeck, Martine Culty

**Affiliations:** Department of Pharmacology and Pharmaceutical Sciences, Alfred E. Mann School of Pharmacy and Pharmaceutical Sciences, University of Southern California, Los Angeles, CA, United States

**Keywords:** Sertoli cell, endocrine disruptors, mixtures, drugs, spermatogenesis, testis

## Abstract

Sertoli cells play essential roles in male reproduction, from supporting fetal testis development to nurturing male germ cells from fetal life to adulthood. Dysregulating Sertoli cell functions can have lifelong adverse effects by jeopardizing early processes such as testis organogenesis, and long-lasting processes such as spermatogenesis. Exposure to endocrine disrupting chemicals (EDCs) is recognized as contributing to the rising incidence of male reproductive disorders and decreasing sperm counts and quality in humans. Some drugs also act as endocrine disruptors by exerting off-target effects on endocrine tissues. However, the mechanisms of toxicity of these compounds on male reproduction at doses compatible with human exposure are still not fully resolved, especially in the case of mixtures, which remain understudied. This review presents first an overview of the mechanisms regulating Sertoli cell development, maintenance, and functions, and then surveys what is known on the impact of EDCs and drugs on immature Sertoli cells, including individual compounds and mixtures, and pinpointing at knowledge gaps. Performing more studies on the impact of mixtures of EDCs and drugs at all ages is crucial to fully understand the adverse outcomes these chemicals may induce on the reproductive system.

## 1 Introduction

Sertoli cells are a critical somatic cell type of the male gonad that produce signaling molecules playing key roles in fertility and reproduction, by regulating and supporting germ cell development. Highlighted in this review are mechanisms by which Sertoli cells are regulated during male gonad development and their function thereafter, from birth to adulthood (1). Additionally, this review seeks to underscore the consequences of exposing fetal and/or perinatal Sertoli cells to endocrine disrupting chemicals (EDCs) as well as to drugs reported to target Sertoli cells. Chemicals are characterized as EDCs based on their ability to perturb hormonal homeostasis, by either disrupting the production of hormones by endocrine tissues, their metabolism, or their functions by altering hormone receptors on target tissues (2–4). While the term “endocrine disruptor”, often referred to as EDC, was coined in the 1990s by Theo Colborn and colleagues, its definition has evolved over the last decades and varies between scientific societies, regulatory and governmental agencies worldwide (5). The health risk assessment of EDCs has been complicated by the acknowledgement of the complexity of EDC effects. There is now a greater understanding of short- and long-term, as well as transgenerational effects, of secondary effects due to increased susceptibility rather than direct adverse effect on target tissues/cells, the recognition that mixtures might have different effects than their individual components, and the advances in molecular approaches, large databases and bioinformatic tools, that have unraveled complex mechanisms of toxicity within biological targets of EDCs. To date, there is still some controversy on which toxic substance should be categorized as EDC. Several consensus statement articles have addressed this issue, as well as the challenge posed by the low dose and non-monotonic effects of some EDCs, and their regulatory implications (6–8). Among the environmental chemicals targeting Sertoli cells, phthalates, xenoestrogens, and metals such as cadmium, are characterized as EDCs.

Despite their critical role, fewer studies have examined the toxic effects of EDCs on Sertoli cells relatively to those focusing on androgen-producing Leydig cells or germ cells. There is a gap of knowledge on the mechanisms by which toxic substances, including EDCs and their mixtures, create a hostile environment during critical developmental periods of the male gonad and how Sertoli cells contribute to the adverse reproductive phenotypes resulting from EDC exposures. The review first summarizes Sertoli cell functions across development and adulthood, then discusses studies reporting the adverse effects of individual EDCs and mixtures, as well as few drugs, on immature Sertoli cells, looking at possible relationships between Sertoli cell disruption and outcomes on testis and male reproduction. The review illustrates the scarcity of publications on the topic, further highlighting the importance of performing more studies to identify potential targets and/or mechanisms disrupted in Sertoli cells during key developmental periods by these toxicants.

### 1.1 Mechanisms regulating Sertoli cell development and function in the developing gonad

#### 1.1.1 Fetal Sertoli cells and their role in driving fetal testis formation

The establishment of Sertoli cells from somatic cell lineage is initiated by the Sry gene, encoding for the transcription factor Sex Determining Region Y (SRY) (9). The indispensable and remarkable role of SRY is demonstrated by the existence of XY humans with frameshift or single base mutations in Sry gene who develop as phenotypic females, whereas the significant expression of Sry gene in an XX individual, mainly due to abnormal Sry translocation to a X chromosome, leads gonads to develop along the male sexual axis (10, 11). These data indicate the importance of SRY in directing gonadal somatic progenitor cells toward the Sertoli cell fate since its absence or dysfunction causes cells to differentiate into granulosa cells along the ovarian pathway. Newly formed Sertoli cells express SRY which targets SOX9 expression toward testis formation (9). SOX9 and fibroblast growth factor 9 (FGF9) communication during early gonad development regulates testis morphogenesis (12). In addition, Prostaglandin D2 (PGD2), a small eicosanoid produced through an enzymatic cascade including cyclooxygenase and PG synthase enzymes from the initial precursor arachidonic acid, induces SOX9 expression in an autocrine fashion within Sertoli cells, that represses the ovarian pathway during male sex determination. The establishment of Sertoli cell lineage in the gonads allows for the organization of testis cords, testis-specific vascular patterning, and appearance of other somatic cell types: Leydig cells, peritubular myoid cells (PTMs) (9).

The appearance of other somatic cell types including Leydig and PTM cells depends on a specific threshold number of Sertoli cells and leads to the compartmentalization of the testis into the testis/seminiferous cords, where spermatogenesis will take place at puberty, surrounded by a basement membrane and either a single (rodent) or multiple (human) layer of PTM cells, and the interstitium for androgen production (9). Fetal cord formation localizes Sertoli and germ cells inside the cords, while Leydig cell clusters, blood vessels, hematopoietic and other somatic cell types are found in the interstitium. Multiple factors contribute to inducing endothelial cell migration for testis vasculature formation, including fibroblast growth factor 9 (FGF9), platelet-derived growth factor (PDGF) A, B (PDGFB), and C (PDGFC), bone morphogenetic protein (BMPs), and anti-Mullerian hormone (AMH), most of them produced by Sertoli cells (1, 9). Sertoli cell-derived hedgehog ligand desert hedgehog (DHH) signaling has been shown to specify Leydig cell lineage, impact testis cord formation, and regulate fetal Leydig cell differentiation (9, 13–15).

During fetal development, newly formed Sertoli cells regulate fetal germ cell development. At around embryonic day (E) E12.5 in mice, Sertoli cells are competent to inhibit the retinoic acid (RA) pathway by expressing the RA-degrading enzyme CYP26B1, when combined with primordial germ cells (PGCs). This process is critical to suppress meiotic entry in male germ cells, while upregulating male germ cell markers that ensure male germ cell differentiation (16). However, this property is lost by E15.5 and E18.5, when Sertoli cells no longer express enough CYP26B1 for degrading RA and suppressing meiotic entry. A second wave of Sertoli cell proliferation and expansion (17) is signaled by fetal Leydig cells to elongate testis cords (9).

#### 1.1.2 Neonatal and immature Sertoli cell, regulation of proliferation

The regulation of Sertoli cell proliferation begins during the fetal to early postnatal period, continuing into the late neonatal period and stopping before puberty, depending on the species (18, 19). Key factors that stimulate Sertoli cell proliferation and development of the male gonad are follicle-stimulating hormone (FSH), insulin-growth factor family (IGF-I and IGF-2), activins, inhibins, and the release of specific cytokines (18). FSH is important in Sertoli cell proliferation during fetal and early postnatal period, whereas FSH regulates differentiation after cessation of mitosis at puberty (18–20). FSHR expression in males is solely found in Sertoli cells (21). Earlier studies investigated immature Sertoli cell proliferation by treating isolated rat Sertoli cells with FSH, which stimulated MEK-1 activation leading to downstream phosphorylation and nuclear relocation of ERK1/2. In turn, cyclin D1 (CCND1) is upregulated to promote cell cycle progression and sustain proliferation (22, 23).

The insulin family of growth factors is involved in growth, metabolism, and reproduction, and can be found during early development in proliferative Sertoli cells (18). IGFs bind to IGF receptors that are coupled to downstream signaling pathways such as that of phosphatidylinositol 3-kinase (*PI3K*)/protein kinase B (*AKT*) *via* the Insulin Receptor Substrates (IRS1-4), adaptor proteins. IRS2 positively mediates IGF-1 receptor signaling in neonatal Sertoli cells for regulating proliferation, involving paracrine signaling to PI3K/Akt (24) and ERK1/2 pathways (25). Of the transforming growth factor (TGF) β superfamily, activins and inhibins are gonadal peptides that play a role in the regulation of Sertoli cell proliferation (18). Activins include activin A (βAβA) and activin B (βBβB), with activin A being mainly produced and secreted by fetal Leydig cells during fetal testis development. Secretion of activin A by Leydig cells is shown in human fetal testis to increase Sertoli cell proliferation directly (26). A significant decrease of activin A concentration is observed in Sertoli cells as they progress to their mature, non-proliferative state (27). Inhibins, specifically Inhibin B, may ultimately play a supportive role in activin A-induced Sertoli cell proliferation (18). Additionally, Sertoli cells secrete specific cytokines, such as two types of Interleukin 1 (IL-1), IL-1α and IL-1β, which were shown to increase DNA synthesis and Sertoli cell number *in vitro*, suggesting a role in Sertoli cell proliferation (28). Additionally, data suggests tumor necrosis factor (TNF) α as a positive regulator of Sertoli cell proliferation (29).

Factors that inhibit or cause cessation of Sertoli cell proliferation to initiate Sertoli maturation include androgens, thyroid hormone, estrogens, and retinoic acid (RA) (18). Androgen production and modulation is key to male fertility and spermatogenesis. Androgens diffuse through the plasma membrane to bind androgen receptor (AR) in the cytoplasm, where it is sequestered by heat shock proteins in the absence of ligand. AR is expressed post-proliferation in Sertoli cells, suggesting a role in cell differentiation (18). The critical timing of AR signaling in Sertoli cells and its role in their maturation was demonstrated with a transgenic conditional mouse model (TgSCAR) in which AR was prematurely expressed in Sertoli cells, triggering androgen-driven processes such as lumen formation too early and shortening the time to spermatid formation, eventually reducing postmeiotic development and testis size (30).

Thyroid hormone (TH) including triiodothyronine (T3) has been shown to inhibit proliferation and stimulates differentiation of culture neonatal Sertoli cells (31). TH has a significant role in Sertoli cell maturation based on extensive evidence showing a negative regulation of Sertoli cell proliferation (32–35). Inhibition of FSH-stimulated Sertoli cell mitosis is mediated by TH and its inhibitory effect is supported by increased expression of cell cycle inhibitors p21Cip1 and p27Kip1 (36). TH may control metabolism *via* AMPK pathway, contributing to its effects in Sertoli cell maturation, as shown by recent reports of AMPK activation reducing FSH-stimulated Sertoli cell proliferation (37). Such interactions between two endocrine tissues are important to note, as they further imply that EDCs affecting one endocrine tissue could have indirect effects on another endocrine tissue.

Initial studies of the regulation of Sertoli cell proliferation showed that *in vivo* administration of estrogen in rats reduced Sertoli cell number (38). A study showed that blocking estrogen receptors (ERs) with the non-specific ER antagonist ICI 182, 780, as well as the aromatase inhibitor letrozole, increased porcine Sertoli cell proliferation, supporting the idea that estrogens halt Sertoli cell proliferation (39). Estrogen receptor (ER) α (ESR1) is also shown to be higher at the proliferative state of Sertoli cells, while ER β (ESR2) increases with age during the differentiation of Sertoli cells. Studies suggest that estrogens are involved in both proliferation and maturation of Sertoli cells depending on the estrogen receptor isoform present (18). The ratio of ERα and ERβ is physiologically important to determine the end of Sertoli cell proliferation and start of cell differentiation (40). Retinoic acid (RA), specifically all-trans RA (atRA), has been studied for its effects in cessation of Sertoli cell proliferation. AtRA presence led to increased expression of p21CIP1 and p27KIP1 which are major players in cell cycle arrest and differentiation of Sertoli cells (36).

#### 1.1.3 Interaction of Sertoli cell with germ cells, a role in gonocyte proliferation and migration

It is known that gonocyte proliferation occurs while Sertoli cells are actively proliferating during fetal and neonatal phases. Mouse and rat gonocytes proliferate first around gestational day (GD) GD14 until GD16, followed by quiescence until they resume proliferation, at three days after birth in rat and one or two days earlier in mouse (41–43). Gonocyte proliferation occurs simultaneously to their migration from the center of the seminiferous cords to the basement membrane at the periphery of the cords (43). Sertoli cells were observed to complete proliferation by postnatal day (PND) 17 in mice (41) based on the absence of [^3^H]thymidine-labelling at that age and in PND20 rats when the presence of thyroid hormone regulates maturation initiation in Sertoli cells (31). The study of Sertoli cell proliferation in fetal and postnatal rats has shown that these cells have proliferative capacity through PND 21, when [^3^H]thymidine-labelling was no longer detected (44).

Gonocyte markers have not been specifically identified to signify induction of resumption of mitotic cell cycle progression, but some factors appear to be associated with quiescence. In mouse testes, gonocytes with increased expression of retinoblastoma 1 (Rb1) and cyclin D kinase inhibitors p15, p16, p21, and p27 proteins are found to coincide with the quiescent period (45). Initiation of gonocyte population out of quiescence and resumption of cell cycle progression is regulated by Sertoli cells proliferative state. A mouse conditional knockout of Activin A, member of TGFβ superfamily, suggests induction of cell cycle arrest modulated by Rb1 and p21 expression (46). Activin A and TGFβ-1 expression significantly decrease after birth, which coincides with resumption of gonocyte proliferation (45, 47). During neonatal development, fibroblast growth factor 2 (FGF2) and leukemia inhibition factor (LIF) are secreted by Sertoli cells as paracrine signals for synergistic stimulation of gonocyte survival (48, 49), while neonatal gonocyte proliferation is regulated by a combination of PDGF-BB and 17β-estradiol (43, 50).

Gonocyte migration and expansion have been shown to be impaired in testes from knockout mice for Doublesex- And Mab-3-Related Transcription Factor 1 (DMRT1), a gene involved in testis development and Sertoli cell differentiation, suggesting some influence in Sertoli-secreted DMRT1 on gonocyte activities (51). Additionally, the Sertoli cell-secreted transcriptional regulator SIN3A was shown to support the transition of a subset of gonocytes to undifferentiated spermatogonia (52). Moreover, Sertoli cells release selective cytokines, including KIT ligand (KITL), platelet-derived growth factor (PDGF), and C-X-C motif chemokine 12 (CXCL12) that influence the migratory behavior of gonocytes (43). KITL deficiency in mice led to impairment of adhesion between gonocytes and Sertoli cells. Studies in mouse testis at PD 1-5 showed that PDGF inhibition led to an increased number of centrally located gonocytes and apoptotic cells (42). Yang and Oatley hypothesize that CXCL12 has a regulatory role on gonocyte migration, based on CXCL12 expression being significantly reduced in Sertoli cells that are SIN3A-deficient, concomitant with an apparent disruption of gonocyte migration (45).

During germ cell development, gonocytes that failed migration require apoptosis (43, 53). Manku and Culty investigated the effects of inadequate gonocyte differentiation and formation of testicular germ cell tumor (TGCT), reporting that differentiating gonocytes and spermatogonia had upregulated pro-apoptotic genes *Gadd45a* and *Cycs* compared to non-differentiating gonocytes (53). This highlights the important role of the phagocytic activity of Sertoli cells for removing residual gonocytes that failed to migrate toward basement membrane (45). This is true also in the pubertal and adult testes, in which the maintenance of normal spermatogenesis relies on Sertoli cells’ phagocytic activity (54). Apoptotic germ cells and residual bodies are phagocytized by Sertoli cells, which maintain the physical and environmental support needed for spermatogenesis (54, 55). A study by Yokonishi et al. replaced an existing Sertoli cell population in male mice with fresh pluripotent Sertoli cells xenografts and showed improvement of spermatogenesis by an increase in spermatogonial survival (56).

### 1.2 Mature Sertoli cell and terminal differentiation

Two waves of Sertoli cell proliferation are known to occur throughout its lifespan: firstly, during late fetal and early neonatal life dividing more than fivefold, and secondly just before puberty in which Sertoli cells grow more than twofold (44, 49). Androgen receptor (AR) expression in Sertoli cell signifies maturation of the cells after proliferation and their responsiveness to androgen during spermatogenesis (57). Sertoli cells acquire AR expression post-differentiation, at the onset of puberty (19). Willems et al. underlined the importance of AR in Sertoli cell using AR ablation models which caused abnormal Sertoli cell maturation and germ cell development (58). Sertoli cell androgen receptor knockout (SCARKO) model studies further showed AR importance in Sertoli cell maturation, demonstrated by increased permeability of the blood-testis-barrier (BTB), and evidence of a cell-autonomous activation of AR, important for normal Sertoli cell function (57, 58).

Sertoli cells undergoing differentiation from fetal into a more mature stage can be identified by specific markers and transcription factors such as FSH, AMH, SRY (male gonad), NR5A1 (SF1), GATA4, WT1, SOX9, and DMRT1. During Sertoli cell development, NR5A1, GATA4, and WT1 are seen as major transcription factors important in directing fetal somatic cells to fetal Sertoli cell fate (59, 60). SOX9 is known to be expressed throughout the lifetime of a male rat, albeit more strongly in the prenatal period, followed by a large decrease after birth (61). Nonetheless, SOX9 likely plays a significant role in adult germ cell differentiation as it is still expressed in adult Sertoli cells. DMRT1 has been shown to be important for testis differentiation in most mammalian species (51). Deleting murine DMRT1 causes Sertoli cells to over-proliferate in the testis as well as induces germ cell death, possibly related to a defect in Sertoli cells.

The role of AMH in Sertoli cell fate has been thoroughly investigated. Based on data collected from mice in the late perinatal period (PND 0-7), AMH was found to differentially regulate Sertoli cell development depending on its concentration (62). Indeed, AMH at low concentrations promoted Sertoli cell proliferation, whereas high concentrations of AMH induced apoptosis. The expression of proapoptotic proteins was upregulated, including cleaved caspase-3 and Bax in the presence of AMH with a decrease of the anti-apoptotic protein Bcl-2. In the presence of higher AMH expression, stem cell factor (Scf) which regulates Sertoli cell development, was also increased. The authors concluded that the timely expression of AMH and its receptor AMHRII in spermatogenesis may act in a paracrine or autocrine manner during testicular development. More recently, AMH secreted by Sertoli cells was shown to play a structural role in the formation of mouse testis (63). Mice positive for Amh-Cre and Diphtheria Toxin A (DTA) were used to generate offspring with Sertoli cell-ablated AMH, which were found to present disruption of testis cords and loss of germ cells. Mislocalization and reduction of α-smooth muscle actin (SMA)-labeled peritubular myoid (PTM) cells were also observed in Sertoli cell Amh-Cre; DTA mice. While it is known that there are markers to identify Sertoli cells at the mature and differentiated state, questions regarding the dynamic state of Sertoli cell and whether it is terminally differentiated have come up (17, 64).

### 1.3 Role of Sertoli cell in primary undifferentiated and differentiating spermatogonia

Spermatogonia differentiation happens alongside the maturation and differentiation of Sertoli cells. The repression of c-KIT expression in gonocytes induces the transition to undifferentiated spermatogonia. Re-establishment of c-KIT expression in undifferentiated spermatogonia leads to transition into differentiated spermatogonia (45, 65). In the presence of KITL expression, a transition from spermatogonial stem cells (SSCs) to differentiated c-KIT-positive spermatogonia occurs. FGF2, glial cell derived neurotrophic factor (GDNF), and CXCL12 are secreted by Sertoli cells that induce a response in undifferentiated spermatogonia to 1) translationally repress c-KIT expression and 2) transition to differentiated spermatogonia (65). GDNF is negatively regulated by NOTCH1 signaling, likely to balance stimulation by FGF2 and FSH in Sertoli cells causing gonocytes exit from quiescence (66). RA secreted by Sertoli cells induces the progression to undifferentiated spermatogonia, a process initially observed in vitamin A-deficient rats and mice, with testes unable to proceed with normal spermatogenesis until RA was reintroduced (67). The concept of an RA intermediate produced by Sertoli cells during the transition from gonocytes to differentiating spermatogonia is becoming increasingly accepted as it was only confirmed within the last decade, based on paracrine communication between Sertoli cells and the transitioning gonocytes (45).

The mature Sertoli cell is known for its impact on tubular architecture, peritubular myoid cell fate, and adult LC number (68). Gap junctional protein (GJA1) which is also known as Connexin 43 (CX43) is vital in Sertoli cell differentiation, but not for spermatogonia cell maintenance/self-renewal (69). Recently, mTOR signaling was shown to be involved to during PI3K/Akt signaling of spermatogenesis and regulates GJA1 within Sertoli cells (70).

### 1.4 Blood-testis-barrier and immune privilege

Sertoli cells and the formation of the BTB create a significant defense mechanism in the testis (71–73). The initial formation of the BTB occurs during puberty and two distinct sections form, based on a division of the seminiferous epithelium by tight junctions (TJs) along the basal region of Sertoli cells into adluminal and basal compartments (1). The BTB require CX43 (or GJA1) expression to maintain normal development and regulation of its dynamics (69, 74). Adult Sertoli cells lie at the basal lamina of seminiferous epithelium, in which BTB are comprised of not only TJs but also of basal ectoplasmic specializations (ES), desmosomes, and gap junctions (GJs) (72). Occludin, claudins-3, -5, -11, zonula occludens (ZO) -1, -2, and -3 and junction adhesion molecules A and B (JAM-A and JAM-B, respectively) are some of the major junction proteins which bridge communication across the BTB (75). Occludin and claudin-11 junction proteins are most important in the maintenance of barrier integrity (1). Androgens not only play a major role in Sertoli cell maturation but also mediate the formation of newly formed Sertoli cell TJs by regulating Claudin-3 expression and maintaining TJs structure, possibly involving Claudin-13, and noncanonical tight junction protein 2 isoform (Tjp2iso3) (76).

The BTB is absent in perinatal and juvenile periods, although some of its junctional proteins are already observed in low levels at these ages, but not organized as they become during puberty to form the BTB, which provides germ cell protection from immune cells (1). Studies elucidating the compartmentalization of immune cells from auto-immunogenic cells, ultimately showed that germ cells are sequestered from immune cells including macrophages, T cells, and dendritic cells *via* one (rodent) or more (human) layers of peritubular myoid cells (72, 77). Therefore, a tight control of keeping auto-immunogenic germ cells physically away from immune cells is established by the selective BTB between Sertoli cells and myoid cells (72). Altogether, the studies cited above depict the BTB as a plastic and highly dynamic structure, where the interaction between signaling molecules such as the focal adhesion kinase FAK and the SRC Proto-Oncogene, Non-Receptor Tyrosine Kinase (SRC) kinase, their regulation and interaction with TJ proteins and actin filaments, participate to the functional remodeling of the BTB, allowing the movement of germ cells, while insuring its essential barrier function.

Immune modulation by Sertoli cells contributes to the immune privilege of the adult testis (72). Recent review of Sertoli cell contributions to the immune privileged testis by Qu et al. (2020) include inter-Sertoli cell junctions, Fas ligand, Activin, IGF-1, epidermal growth factor, TGFβ, and lipocalin (78). Induction of regulatory T cells (Tregs) by Notch/JAGGED1 signaling and stimulation of peripheral conversion of CD4^+^FOXP3^-^ T cells to functional CD4^+^FOXP3^+^ Tregs in the presence of mouse Sertoli cell conditioned medium has been shown (79). Most recently, Kaur et al. (2020) used neonatal pig Sertoli cells to suggest a Sertoli cell immunosuppression mechanism *via* upregulated CD4 and CD8 Tregs and other immunoregulatory factors to improve survival of xenografts (80).

Due to their ability to produce immunomodulatory factors and maintaining the immune privilege status of the seminiferous tubules, Sertoli cells have recently been successfully used to provide immune protection to pancreatic islets xenotransplants and proposed as novel therapeutic tools in a number of diseases (81), broadening the scope of these cells beyond reproduction.

## 2 Consequences of exposing Sertoli cells to endocrine disrupting chemicals

EDCs represent a particular type of toxic substances, including natural and manmade chemicals, that target the endocrine system at the developmental and/or functional levels from fetal to adult life. A major target of EDCs is the developing reproductive system (82). The testis is a complex endocrine tissue regulated not only through the hypothalamus-pituitary-gonadal axis *via* pituitary hormones and negative feedbacks from Leydig and Sertoli cells, but also by interactions with other endocrine tissues, such as the adrenals (83). The detrimental effects of EDCs on fetal and perinatal Sertoli cells can have lifelong adverse consequences on testicular functions, primarily spermatogenesis, and male fertility. Changes in Sertoli cell proliferation rates and differentiation, cytoskeleton, BTB and Sertoli cell-germ cell adhesion, have been observed in response to EDCs (73, 84–87). Gao et al. state in their expert opinion that despite knowing that toxicants such a carbendazim and phthalates act on microtubule-based Sertoli cell cytoskeleton, while PFOS, BPA, and cadmium act on actin-based Sertoli cell cytoskeleton, the mechanism by which these effects occur is still largely unanswered. This section will provide examples of EDCs acting on Sertoli cells.

### 2.1 Cadmium

Cadmium is a chemical encountered by humans *via* occupational and environmental exposures. Cadmium-induced testicular injury was first observed in 1957 by Parizek and is known for its effects as an endocrine disruptor (85, 88). The effects of cadmium on Sertoli cell BTB integrity have been thoroughly investigated (84). Cadmium was found to induce human fetal germ cell apoptosis in organ cultures treated for 4 days with 1 and 10 μM Cadmium, likely *via* direct effect on germ cells, since neither fetal Leydig cell-produced testosterone nor the number of AMH-positive Sertoli cells were affected, suggesting that human fetal Leydig and Sertoli cell functions were not impaired by Cadmium (89). However, an *in vivo* study examining the effects of cadmium on fetal rats to a single low dose of Cadmium, showed that fetal Leydig cells and Sertoli cells were affected by Cadmium, as shown by reduced testosterone production, lower Leydig cell numbers, and decreased protein expression of several Sertoli cell markers, including desert hedgehog (DHH), AMH, and follicle-stimulating hormone receptor (FSHR) in fetal testes (90). These divergent data could be due to the difference between using *in vivo* vs organ culture models, or to the fact that fetal rat testis is more sensitive to Cadmium than fetal human testis. Such difference between species is reminiscent of a study comparing the effects of phthalates on human and rat fetal testis xenografts, in which rat Leydig cells were sensitive to phthalates, but not human Leydig cells (91).

The adverse effect of Cadmium on Sertoli cells is demonstrated by its disruptive effects on the BTB (84, 85). Several signaling molecules playing a role in BTB that are disrupted by Cadmium have been identified, including MAPK proteins i.e. ERK1/2 and MEK1/2 and focal adhesion kinase (FAK) proteins, and their phosphorylation status, important in regulating signaling to TJ proteins occludin and ZO-1 (84). More recently, Zhou et al. found that, in adult mouse testes, autophagy in Sertoli cells is crucial for mediating protection against Cadmium-triggered germ cell apoptosis (92). It is noticeable that most studies focus on adult exposure to Cadmium and the mechanisms altered within adult Sertoli cells by Cadmium, while there are minimal studies observing the impact of fetal and/or postnatal exposure to Cadmium on the formation and functioning of BTB later in life, which would suggest a Cadmium-driven injury of developing rather than mature Sertoli cells. Such delayed effect could be the result of either a direct effect of Cadmium on immature Sertoli cells, as suggested by the studies on fetal testis mentioned above, or the disruption of another testicular function affecting indirectly Sertoli cells.

### 2.2 Bisphenol A (BPA)

BPA is an estrogenic EDC suggested to have detrimental reproductive effects following fetal exposure. Most human exposure to BPA occurs *via* housewares made of polycarbonate plastics, medical devices, and packaging. BPA exposure during fetal development has been shown to inhibit rat Sertoli and Leydig cell differentiation, as shown by the downregulation of AMH production by fetal Sertoli cells and lower testosterone production, indicating that BPA impaired Sertoli and Leydig cell functions, further jeopardizing the development of the male reproductive tract (93). In contrast, organ cultures of GD15.5 fetal mouse testes exposed to BPA for 5 days showed a slight increase in fetal Sertoli cell number and gene markers including *Amh*, *Sox9*, and *Wt-1*, leading the authors to propose that BPA might have positive effects on mouse fetal Sertoli cells. However, fetal germ cell number and gene markers, as well as Leydig cell markers were decreased. Thus, the positive effect on fetal mouse Sertoli cells postulated by the authors might have instead resulted in the disruption of the ratio between Sertoli and germ cell numbers, abnormal levels of Sertoli-produced signaling molecules involved in fetal germ and Leydig cell regulation. In such case, the toxic effect of BPA on mouse fetal testis could be driven in part by an unbalance in factors regulating the communication between the three cell types (94). Exposing explants of rat and human fetal testes to BPA showed that AMH production in fetal Sertoli cells was not affected by BPA exposure, indicating that fetal Sertoli cells were not targeted by BPA, whereas BPA exerted antiandrogenic effects resulting in reduced testosterone production in both species (95, 96). Thuillier and colleagues reported transient alterations of MAPK pathway in neonatal testes following *in utero* exposure to BPA or genistein, including increased expression of Raf1, and ERK1/2 proteins in Sertoli cells, but these changes were no longer observed in adult testes (97). All together, these data suggest that fetal exposure to BPA might increase mouse fetal Sertoli cell markers or signaling molecules in rat neonatal Sertoli cells but had no effect on human fetal Sertoli cells, suggesting species specific differences in BPA effects.

Several studies looked at BPA effects in immature Sertoli cells. Rossi and colleagues examined the effects of BPA on PND7 mouse primary Sertoli cells, reporting that exposure to 0.5 µM BPA increased the endocannabinoid (eCB) signaling, a family of lipid signal molecules that binds the cannabinoid receptors, previously shown to control spermatogenesis and sperm fertilizing ability (98). Because FSH modulates eCB signaling in immature Sertoli cells, the authors proposed a possible deregulation of FSH signaling by BPA. BPA also increased the production of the Sertoli cell functional marker inhibin B, but did not affect transferrin production, another marker of Sertoli cell function. Zhang and colleagues performed single-cell RNA sequencing (scRNA-seq) analysis of human juvenile testis determining germ cell-somatic cell interactions. They identified maturation markers expressed in Sertoli cells that regulate sperm maturation and reported that BPA affected the testis microenvironment that contributes to germ cell maturation, as a possible mechanism of BPA reproductive toxicity mechanisms (99).

The immature mouse Sertoli cell model TM4 was used to determine whether alternative structural analogs of BPA are safer than the legacy chemical BPA that they intend to replace, using high-content imaging analysis. Morphometric and functional parameters, including numbers of Hoechst-stained nuclei, lysosomes, lipid droplets, mitochondria and oxidative stress that were used to rank cytotoxicity, indicated that the alternative chemicals were more potent than the legacy chemical in inducing Sertoli cell toxicity (100). Another study performed on TM4 cells examined the impact of exposures to EDCs such as BPA, pesticides, and personal care product components, on the expression of TJ and GJ proteins. Using a scalpel loading-dye transfer technique to study gap junction morphological changes, they observed that BPA exerted a low inhibitory activity on testicular gap junctional intercellular communication that was increased with longer time of exposure, leading to altered phosphorylation status and translocation of the GJ protein Cx43, suggesting BPA ability to dysregulate testes development *via* its action on gap junctional intercellular communications in Sertoli cells (101). Another study using TM4 identified the androgen signaling pathway as a BPA target, based on a combination of end-points, showing that BPA treatment decreased TM4 cell proliferation, concurrently with inhibiting the interaction of the amino- and carboxyl-terminal regions (N/C) ends of androgen receptor (AR), and enhancing AR interactions with the co-repressors NCoR (Nuclear Receptor Co-Repressor) and SMRT (Silencing Mediator Of Retinoic Acid And Thyroid Hormone receptor) (102). The authors proposed a role of BPA as anti-androgenic, besides its well-established estrogenic effects, in TM4 Sertoli cells, resulting in the reduction of cell proliferation. However, these results suggest that the TM4 mouse cell line, generated from PND11-13 mice, might not represent a genuine immature Sertoli cell model, since the proliferation of immature Sertoli cells is inhibited by androgens (18).

In another study on TM4 Sertoli cells, BPA was found to have a biphasic effect, with micromolar BPA levels causing inhibition of cell proliferation while nanomolar BPA levels stimulated energy metabolism, such as higher ATP concentrations and greater mitochondria activity (103). Qi et al. (2014) attributed BPA-induced apoptosis of PND 18-22 rat Sertoli cells to the Fas/FasL and p38/JNK pathways (104). Additionally, *in vitro* study of primary Sertoli cells exposed to EDC mixture of phthalate MBP and BPA exerted disruptive effects on tight and gap junctions, reduced androgen receptor and junction protein expression, to result in overall disturbance of BTB (86). Having conducted phalloidin and hematoxylin eosin staining of Sertoli cells treated with MBP and BPA showed a loss of cell number and adhesion between Sertoli-Sertoli cell interaction.

### 2.3 Phthalates

Phthalates (phthalic acid esters) are another group of EDCs that causes disruption to male reproductive function, found in consumer products such as shampoo, cosmetics, hairspray, food packaging and other consumer products. Phthalates, also known for their use as plasticizers, include di(2-ethylhexyl) phthalate (DEHP), metabolized to bioactive MEHP, as well as di(n-butyl) phthalate (DBP) that breaks down into mono(n-butyl) phthalate (MBP) (105–107). The deleterious effects of these phthalates on fetal testis function are well documented, including decreased androgen production, and some effects on Sertoli cells, such as decreased secretion of Androgen Binding Protein (ABP) and induction of morphologic changes in prepubertal rat Sertoli cells by MEHP (106, 107). Additionally, FSH receptor signaling was altered in immature Sertoli cells exposed to MEHP (108). In cultured human fetal testes, *Amh* mRNA expression as well as germ cell number were reduced by MEHP treatment, with an increase in germ cell apoptosis detected by caspase-3-positive immunostaining (109).

Human studies, such as that conducted on the mother-child Hokkaido Study Sapporo Cohort, have also established a strong correlation between maternal phthalate exposure and abnormal Sertoli cell function in their infants (110). In this study, the concentration of MEHP, major bioactive metabolite of DEHP, was determined in the blood of pregnant women, while a panel of reproductive hormones were measured in cord blood to determine Sertoli and Leydig cell functions in the infants. The study showed that the Sertoli cell marker Inhibin B was decreased in a dose-dependent manner with increasing maternal levels on MEHP, identifying perinatal Sertoli cells as phthalate targets. However, since Leydig cell markers were also decreased in cord blood, one cannot conclude if Sertoli cells were primary or secondary targets of the phthalate in this case (110). Although such studies are essential for the validation and extrapolation of findings in animal models to humans, they also illustrate some of the limitations of *in vivo*, organ and organoid studies, which reflect better real exposure conditions at the organism or tissue levels but complicate the determination of primary vs secondary target cells of EDCs, based on physiological parameters. MEHP was found to induce oxidative stress in prepubertal rat Sertoli cells, an effect that was attenuated by combining MEHP with a low dose of genistein, suggesting a protective effect of genistein (111).

Elucidating the mechanisms by which phthalates cause dysfunction in testes is critical. Exposure of fetal and postnatal mice to DEHP at 2mg/kg/day and above, was found to prevent Sertoli cell differentiation by downregulating genes involved in Sertoli cell differentiation such as Sox9, FGF9, and DMRT1 (112). Furthermore, the sex determination pathway, Gadd45g → Gata4/Fog2 → Sry → Sox9 → FGF9 was altered in the same treatment conditions. A more recent study of MEHP exposure in mouse Sertoli cell line TM4 was performed to assess transcriptomic alterations (113). Based on high-throughput sequencing of RNA collected from TM4 cells, genes that were differentially expressed associated with the Gene Ontology (GO) term “extracellular region” when treated with high and low MEHP doses. Most recently, Ma et al. (2020) found that DBP causes abnormal proliferation of Sertoli cells in prenatal stage of male mice at low concentrations, *via* ubiquitination of interleukin-1 receptor-related kinase 1 (IRAK1) (key proliferation-related protein) by downregulating the E3 ubiquitin ligase Pellino 2 (Peli2) (105). They also suggested the activation of the intrinsic apoptotic pathway to increase apoptosis of TM4 cells when exposed to 10mM MBP.

### 2.4 Effects of xenobiotics on Sertoli cell apoptosis, cytoskeleton, and differentiation

Humans are exposed to chemical compounds that act as endocrine disruptors from agricultural farming practices such as applying pesticide/herbicide to crops, waste accumulation, and burning residues. The pesticide Methoxychlor, a chemical with estrogenic/antiandrogenic effects, given orally to rats during the perinatal/juvenile period was shown to reduce the spermatogenic potential *via* reduction of Sertoli cell number (114). Zearalenone is a mycotoxin with a dangerous secondary fungal metabolite that is found in food and animal feed and induces reproductive toxicity. Cell-cycle arrest in the G2/M phase of Sertoli cells isolated from Wistar rats exposed to the mycotoxins induced autophagy *via* PI3K/AKT/m-TOR signaling pathway (115).

Two plant-derived toxins, colchicine and carbendazim were shown to induce histopathology on adult Sertoli cell cytoskeleton (116). The seminiferous epithelium was targeted, causing germ cell sloughing off by either colchicine or carbendazim. This process has been proposed to occur due to a destabilization of Sertoli cell stalk by a lack of microtubule support. Other histopathologies included retained spermatids, seminiferous epithelium vacuolization, seminiferous epithelium atrophy, and enlarged seminiferous tubule lumen in relation to defects in Sertoli cell cytoskeleton and signaling.

Exposure of mice from GD7 to PND21 to the flame retardant deca-bromodiphenyl ether (BDE-209) was shown to disrupt BTB function by damaging the TJ proteins occludin, claudin-11, ZO-1, and F-actin *via* estrogen receptor α (ER-α) signaling (117). Another flame retardant, aroclor1254, impaired BTB by increasing caveolin-1 associated endocytosis of several junctional proteins and enhancing ubiquitination of occluding by E3 ligase Itch (118). TJ proteins were shown to be delocalized from the cell membrane to the cytoplasm in PND20 rat Sertoli cells exposed to glyphosate alone or in Roundup, a common pesticide used in agricultural practice (119). However, when glyphosate or Roundup 3 Plus (GBHs) was given to pregnant mice from GD10.5 to PND20, the deleterious effects of glyphosate and GBHs were to reduce the number of undifferentiated spermatogonia, while only glyphosate decreased testosterone levels, and perinatal Sertoli cells were rather unaffected (120). The two studies suggested different species susceptibility to glyphosate.

Furthermore, transgenerational changes in rat Sertoli cell epigenome and transcriptome were associated with the onset of testis disease (121). Gestating female rats were transiently exposed to the environmental toxicants dichlorophenyltrichloroethane (DDT) or fungicide vinclozolin at the period of fetal gonadal sex determination and later bred to analyze the F3 generation (i.e., great-grand-offspring) for possible effects on transgenerational Sertoli cell genetic profiles. DNA and RNA obtained from purified Sertoli cells in PND20 male testis of F3 generation rats showed transgenerational alterations in DNA methylation, noncoding RNA, and gene expression following the treatment of F0 females with DDT or vinclozolin and exposure of the F1 fetuses they carried, compared to control rats. These transgenerational alterations in Sertoli cells were suggested to be critical factors in developing transgenerational testis pathologies and male infertility.

### 2.5 Perfluorooctane sulfonate (PFOS) or perfluorooctanoic acid (PFA)

Another well-known EDC class is that of per- and poly-fluoroalkyls substances (PFAS), which includes perfluorooctane sulfonate (PFOS) or perfluorooctanoic acid (PFOA). PFOS is commonly found in paints, textiles, general cleaning products, and other consumer products. *In vitro* studies showed that PFOS perturbs male rat BTB function by affecting F-actin organization *via* p-FAK-Tyr407 (122). PFAS are known to exert detrimental effects on the BTB of adult human and rat Sertoli cells, mechanistically similar to those of cadmium and BPA, described as inducing testis injury *via* action on Sertoli cell actin- and/or microtubule-based cytoskeletal support (123). Investigators proposed that these chemicals acted by disrupting the F-actin microfilaments of the BTB, leading to the loss of support in Sertoli cell adhesion mediated by tight junctions (TJ) and basal ectoplasmic specialization (ES). The affected junctional proteins were occludin, ZO-1, N-cadherin, and β-catenin that were either altered at the expression level or mislocalized (122, 124).

Actin filaments were found to be truncated in BTB due to PFOS exposure. MicroRNA miR-135b, specific to FAK protein, was transfected into Sertoli cells to knock down expression of FAK, which worsened PFOS-mediated Sertoli cell tight junction disruption. PFOS were proposed to mediate Sertoli cell injury by compromising BTB integrity or formation, whereas the effects of PFOA were less serious and mainly limited to the disruption of hormonal pathways in human and rat (125, 126). Moreover, a study comparing the effects of PFOA exposure in fetal and adult rat testis suggested that there were no major effects on the fetal, proliferating, and adult Sertoli cells. Rather, they found reduced steroidogenesis function, increased fetal Leydig cell apoptosis, and PFOA-induced germ cell apoptosis in adult rat seminiferous tubules (127). Taken together, these studies highlighted the difference between PFOS and PFOA in target cell types, with only PFOS acting primarily on Sertoli cells, despite their structural similarities.

### 2.6 Exposure to EDCs in the adult Sertoli cell

The consequences in exposing adult Sertoli cells to EDCs could be different from those of exposing perinatal and juvenile Sertoli cells or targeting fetal development by reducing Sertoli cell proliferative capacity, Sertoli cell number, or spermatogonia population (based on reduced Sertoli cell number). Moffit et al. observed histopathological manifestations that vary in type and occur in a dose-dependent fashion when exposed to 2, 5-hexanedione, carbendazim, and MEHP in adult rat testis (128). Manifestations observed are not limited to but describe change in seminiferous tubule diameter, sloughing, vacuolization, retained spermatid heads, and germ cell apoptosis. Cadmium has also dangerous effects on the BTB in adult rat Sertoli cells, shown to target F-actin organization to impair adhesive Sertoli-Sertoli or Sertoli-germ cell interactions (124). Indeed, BPA and Cadmium *in vitro* treatment of adult human Sertoli cells showed that both EDCs exerted cytotoxic effects by disorganizing F-actin microfilaments, leading to their truncation and depolymerization. β-catenin, ZO-1, and N-cadherin expression levels were not altered, but these proteins were found to be mislocalized in immunostaining imaging with higher doses of Cadmium (>5µM) and BPA (>40µM) (124).

Another environmental toxicant that is prevalent in densely packed metropolitan areas is PM2.5 urban fine particulate matter, which mainly originates from coal combustion and vehicle exhaust. The testes of Sprague-Dawley rats exposed to PM2.5 particles, showed excessive ROS-mediated autophagy that damaged BTB integrity (129). The testes from rats exposed to PM2.5 had increased autophagosome based on transmission electron microscopic imaging. Sertoli cell dysfunction was proposed to be due to decreases in Occludin, Zo-1, and CX43 protein levels, as determined by immunoblot analysis following PM2.5-treatment compared to control, with reduced TJ and GJ integrity. A more recent study of PM2.5 exposure from automobile exhaust reported the induction of oxidative stress *via* MAPK pathway activation. BTB damage was observed in primary Sertoli cell culture, based on increased Sertoli cell apoptosis and decreased expression of Zo-1 and β-catenin, and F-actin disorganization under immunostaining. The authors concluded the BTB damage induced by PM2.5 exposure was regulated *via* a ROS-MAPK-Nrf2 pathway (130).

## 3 Effects of pharmaceuticals on Sertoli cells

Several pharmaceuticals have been found to disrupt Sertoli cell functions as off target effects. The importance of identifying such drugs is evident in view of the multiple and essential roles played by Sertoli cells, from the initiation and regulation of testis development in fetus to the maintenance of spermatogenesis and immune privilege in adult. Some of these pharmaceuticals exert their toxic effects by altering key structures in Sertoli cells, while others impair their hormonal function, such as the production of the nonsteroidal hormones Inhibin B and AMH. The section below presents examples of pharmaceuticals individually altering Sertoli cells.

### 3.1 Non-hormonal male contraceptives effects on the BTB

We are starting this section on pharmaceuticals with the example of non-hormonal male contraceptives that disrupt the integrity of the BTB and Sertoli cell junctions, because they represent an example where Sertoli cell functional disruption is desired rather than accidental. Indeed, the BTB is positioned both as therapeutic target to counter toxicant-induced infertility/subfertility, and as contraceptive target to disrupt spermatogenesis and block sperm formation in a reversible manner (87). The challenge remains to identify an agent that interferes only transiently with Sertoli cell functions and permits the recovery of spermatogenesis in a timely manner, while being also devoid of non-reproductive adverse effects. Currently, several non-hormonal male contraceptives have reached the stage of clinical phases, but none has yet been approved by the FDA, as summarized in the review of Arifuzzaman and colleagues (131). Male contraceptives such adjudin were shown to exert deleterious effects by damaging the BTB, resulting in infertility of adult rats (132, 133). mTORC1/rpS6 signaling complex, previously shown to be upregulated in BTB remodeling, was identified as mediating adjudin adverse effects on the BTB (134). The use of a constitutively active rpS6 mutant in combination with adjudin treatment was found to create a “leakier” BTB, promoting the entry of the nonhormonal male contraceptive into the adluminal compartment in the seminiferous epithelium to induce germ cell exfoliation. A recent article examined the effects of a single oral dose of adjudin at 50 mg/kg on rat testis, reporting that the adverse effect of adjudin on the BTB involved changes in actin dynamics and microtubule associated proteins (MAPs), in addition to disrupting mTORC1/rpS6 and p-FAK-Y407 signaling pathways (135). Although the study confirmed the reversibility of adjudin treatment, it reported long recovery times, from 163 days to recover full spermatogenesis, to 254 days to recover fertility in adjudin-treated rats. This long recovery time in rat, a species where a complete spermatogenic cycle takes around 54 days, and recovery periods of 8 weeks have been reported for toxicants affecting spermatogonia, suggests that the extend of damages afflicted on the Sertoli cell cytoskeletal and junctional proteins by adjudin was extensive (136). Similarly to adjudin, *l*-CDB-4022 acts on Sertoli-germ cell adherens junction proteins to effectively reduce spermatogenic activity (137). *l*-CDB-4022 also induces germ cell loss by activating MAPK pathway to suppress pro-survival factors such as stem cell factor, and inducing pro-apoptotic factor Fas ligand (131). Taken together, non-hormonal contraceptives like adjudin, *l*-CDB-4022, H2-gamendazole, and F5-peptide exemplify the possible therapeutic use of chemicals that target Sertoli-germ cell interactions at BTB junctions as a way to temporarily pause spermatogenesis and control male fertility.

### 3.2 Effects of pharmaceuticals on Sertoli cell proliferation and maturation

A number of pharmaceuticals are known to inadvertently cause male infertility, including drugs exerting adverse effects on Sertoli cells (18). Some examples of drugs affecting Sertoli cells are described below, although in some instances, it is not clear whether Sertoli cells are primary or secondary targets.

#### 3.2.1 Chemotherapy drugs

Few studies have shown effects of chemotherapy drugs on Sertoli cell population, though these drugs are well-studied for its testicular toxicity effects. Additionally, whether the deleterious effects are also observed in the fetal or perinatal period of Sertoli cell population has yet to be characterized. Meroni et al. review some landmark studies of chemotherapeutic agents including cytosine arabinoside and doxorubicin. Though studies did not fully assess whether the Sertoli cell population is at risk during prepubertal exposure to chemotherapy, it has been shown some thirty years ago that intratesticular injection of cytosine arabinoside caused inhibition of Sertoli cell proliferation (138).There is somewhat of a debate on whether Doxorubicin contributes to a decrease in final Sertoli cell number in culture (139, 140), whereas others find germ cells to be primary target (141) with a loss in germ cell number. Additionally, other drugs including cisplatin, doxorubicin, and cyclophosphamide did not affect Sertoli cell number, but there was a significant loss of germ cells in a prepubertal *in vitro* model of mouse testis (142). Most recently, a comparative analysis of cisplatin and carboplatin exposure to prepubertal mouse gonads showed no significant effects on Sertoli cell population (143). Rather, a reduced germ cell number was observed when treated with either carboplatin (0.5µg/ml) or cisplatin (0.05µg/ml) in the testis, suggesting that carboplatin is no less gonadotoxic than cisplatin.

#### 3.2.2 Antiviral agents

Antiviral agents such as nucleoside analogs acyclovir and ganciclovir important in treating infections caused by herpes simplex virus, varicella zoster virus, and other viruses, were observed to decrease testis weight and sperm count in adult animal studies (144). Treating pregnant mice with a high dose of ganciclovir did not alter Sertoli cell number per tubular cross section in their male offspring, but it decreased the number of gonocytes in fetal testis (145). An *in vitro* study using the Sertoli cell line SerW3 treated with either ganciclovir or acyclovir reported that the drugs caused a decrease in GJ protein CX43 expression, suggesting an overall effect on Sertoli cell maturation. Another study exploring the effects of ganciclovir showed a decrease in Sertoli cell proliferation and number which likely related to the decrease in germ cell proliferation and overall number in adulthood (146).

#### 3.2.3 Nonsteroidal anti-inflammatory drugs (NSAIDs)

Another drug class important to examine for potential unintended effects on gonad development is that of NSAIDs. Recently, Ibuprofen was shown to downregulate AMH and SOX9 expression in human fetal Sertoli cell, suggesting decreased Sertoli cell maturation as a result (147). Sharma et al. attribute Selenium (Se) deficiency in maintaining ROS levels to preventing testicular toxicity from prolonged use of Ibuprofen (148). They concluded that supplementation of Se *via* antioxidant enzymes along with Ibuprofen may alleviate Ibuprofen-induced male reproductive toxicity. Acetaminophen, a common analgesic drug, was shown to cause dysregulation to testis development in F1 offspring (149). This was indicated by delayed Sertoli cell maturation and Leydig cells that were hyperplasic in mouse F1 testes.

#### 3.2.4 Metformin

Metformin is an anti-hyperglycemic agent that Type 2 diabetic patients commonly use (18). The expanded use in pregnant women with gestational diabetes mellitus or preeclampsia or polycystic ovarian syndrome has raised some concerns for fetal exposure, as it is known that metformin can cross the placental barrier (150). It has been shown that there is a reduction in Sertoli cell number in fetal life and at birth after *in vivo* administration of metformin in pregnant mice (151). Sertoli cell proliferation is negatively affected by metformin exposure by increasing p21Cip1 levels *in vitro*, decreasing testis weight, and seminiferous tubule diameter (152). Cannarella et al. found that insulin modulates Sertoli cell function by assessing gene expression of Amh, Inhibin B, and AKT473 with or without FSH-stimulation of neonatal porcine cells *in vitro*. Interestingly, their results suggest that hyperinsulinemia may influence testicular dysfunction before puberty. Therefore, hyperinsulinemia onset in childhood should be prevented to preserve fertility (153).

### 3.3 Infertility treatment - hormone therapy

Treatment of male infertility mentioned here include hormone human chorionic gonadotropin (hCG), selective estrogen receptor modulator clomiphene citrate (off-label drug for male infertility), and aromatase inhibitor Anastrazole (off-label drug for male infertility) to offset LH, FSH, and/or testosterone deficiencies. A review by Kilcoyne et al. reported effects of pharmaceutical and environmental exposure with pesticides and pharmaceuticals such as Valproate and Theophylline which highlights effects on testosterone production but minimally affected Sertoli cell population (96).

Ameli et al. highlighted a mechanism by which Clomiphene and hCG exposure of adult Wistar rats acted *via* cation channel sperm-associated protein 1 (CatSper1), cation channel sperm-associated protein 2 (CatSper2), luteinizing human chorionic gonadotropin receptor (LHCGR) and steroidogenic factor 1 (SF1) signaling. Based on treatment with either clomiphene (5mg/kg), hCG (100 IU/kg), or both (5mg/kg and 100 IU/kg respectively), the aforementioned genes were analyzed. CatSper1 and 2, LHCGR, and SF1 were upregulated in hCG-treated rats, while these genes were downregulated by Clomiphene treatment alone. Mechanism aside, the analysis of testicular tissue suggested a reduction in number of Sertoli cells, spermatogonial cells, primary spermatocytes, and spermatid cells with Clomiphene treatment. The authors proposed that while a reduction of these cell populations was observed, they did not see a significant reduction of Sertoli cells specifically. This study concludes that supplementation of hCG in treatment of clomiphene will be neutralized to maintain a normal spermatogenesis process which is otherwise hampered by Clomiphene alone.

Another off-label drug used to treat male infertility specifically in males with a low testosterone to estradiol (T/E_2_) ratio is the aromatase inhibitor Anastrozole (Arimidex), based on the idea that blocking estrogen production may preserve higher levels of its androgen precursor (154). Guercio et al. have reviewed this aromatase inhibitor, which regulates inhibin B expression in Sertoli cells, and the production of testosterone with a focus on childhood and puberty development (155). Shoshany et al. provided some clinical relevance for using anastrazole in oligozoospermic hypoandrogenic men, since improved sperm parameters and T/E_2_ ratio were observed in Anastrazole-treated men (156). They attributed these effects to the inhibition of estradiol formation by anastrozole, which normally stimulates the growth and development of Leydig cells, which in turn act as paracrine regulators of Sertoli cells. While studies regarding male contraception or infertility mention Sertoli cells as possible targets (157), the focus has often been on testosterone production by Leydig cells and spermatogenesis (158). Since Sertoli cells “nurse” the germ cells and interact with Leydig cells and other gonadal cell types, they likely play a role in successful treatments of male infertility.

## 4 Effects of EDC mixtures and EDC-pharmaceutical mixtures on Sertoli cells

Because humans may be concomitantly exposed to multiple EDCs, as well as to pharmaceuticals and EDC mixtures, it has become evident that a thorough risk assessment of exposures to EDCs and to pharmaceuticals with reproductive side effects should consider the possibility that mixtures might not have the same effects as their individual components. There is sufficient evidence of EDCs modifying their respective effects or triggering transcriptome changes or biological responses unique to mixtures, to justify the inclusion of mixtures in toxicology studies (159). However, selecting what mixtures to study, the doses of each chemical, the ages and lengths of exposures, the endpoints to examine, to emulate realistic human exposures and generate data that can be used in risk assessment and recommendations is a difficult task (160).

Co-existing chemicals in the environment, such as metals, xenoestrogens, phthalates, pesticides, and pharmaceutical drugs each have their own mechanisms of action on male reproduction but understanding whether their mixtures could induce further dysregulation is lacking and crucial to investigate. A compilation of published studies observing the effects of mixtures of toxicants, including EDCs and drugs, on Sertoli cells function and/or development is presented in **Table 1**.

**Table 1:** Types of EDC and EDC-drugs mixtures reported to affect Sertoli cells.

EDC/drug mixtures	Treatment type	Sertoli cell molecular/cellular changes; altered phenotypes	Citations
Cadmium and Diazinon (metal + insecticide)	4 wk-old rats; *In vivo*; cadmium (30mg/L) and diazinon (40mg/L) in drinking water for 90 days	Adult Sertoli cell only phenotype in ST and exfoliation of germ cells likely due to damage to Sertoli cells and tight & adherent junctions disruption	Adamkovicova et al., (161)
Genistein and DEHP (phytoestrogen + phthalate plasticizer)	Rats; *in utero* exposure from GD14 to birth; gavage with GEN + DEHP mixture; each at 10 mg/kg/day	decreased Sertoli cell marker gene expression Amh and Wt-1 in PND120 adult rat testes	Jones et al., 2014 (162)
Genistein and phthalate DEHP	Rats; *in utero* exposure from GD14 to birth; gavage with DEHP alone or GEN + DEHP mixture; each at 10 mg/kg/day	caused decreased Sertoli cell marker Abp gene expression in PND6 juvenile rat testes	Jones et al., 2015 (163)
Genistein and phthalate DEHP	Rats; *in utero* exposure from GD14 to birth; gavage with GEN + DEHP mixtures at 0.1 or 10 mg/kg/day	In adult rat testes (PND120): altered morphology of seminiferous tubules. Increased rate of abnormal testicular phenotypes such as Sertoli-cell only; atrophied tubules; germ cell sloughing in lumen	Walker et al., (164)
DBP and Nonylphenol (NP) (Phthalate plasticizer + chemical used in industrial and consumer products)	*In vitro;* isolated primary Sertoli cells from 30 day-old Rat; mixtures of NP (range 0.01–50 µM) and MBP (range 10–20000 µM)	Additive effects on decreased cell viability of Sertoli cells, but no dramatic morphological changes	Li et al., (165)
DBP/MBP and Nonylphenol (NP) (phthalates + chemical used in industrial/consumer processes)	*In vitro*; isolated primary rat Sertoli cells from 9-day-old rats; NP (range 0.01–50 µM) and MBP (range 10–20000 µM).	Synergistic effect on viability and LDH leakage rate (plasma membrane integrity) at lower concentrations, but antagonistic efffect at higher concentrations. Testing 2 mathematical models based on the Loewe additivity (LA) theory and the Bliss independence (BI) theory.	Hu et al., (166)
DBP/MBP and Nonylphenol (NP) (phthalates + chemical used in industrial/consumer processes)	*In vitro*; Sertoli cells isolated from 9-day-old rats; NP (0.1, 1, 10 mM), MBP (0.1, 1, 10 mM), NP + MBP (mixtures at same dose for both). *In vivo:* PND23–35 rats gavaged with DBP and NP as single or mixed compounds	Disrupted structure/function of Sertoli cells and dysregulated hormone levels in serum by exposure to NP and DBP	Hu et al., (167)
Phthalate mixtures: Diethyl phthalate (DEP), diphenyl phthalate (DPP), and dimethyl isophthalate (DMIP)	*In vitro:* primary Sertoli cells isolated from 3-weeks old male mice. *In vivo*: 3-weeks-odl rats exposed by gavage for long-term (45 days)	*In vitro* exposures: Gene expression changes in mouse Sertoli cell markers transferrin, testin, occludin *In vivo:* decreased protein expression of 3β-HSD, connexin-43, and occludin after 45 days exposure. alteration of tight junctions, widening of intercellular space with phthalates.	Kumar et al., (168)
Anti-malaria insecticides: DDT, DDE, DM Industrial pollutant/plasticizer: NP Phytoestrogens: coumestrol, genistein	*In utero*, lactation, then direct exposure of rats up to PND104. exposure to a mixture of DDT, DDE and deltamethrin (DM) (35 mg/kg); to p-nonylphenol (p-NP) (2.5ug/kg); and phytoestrogens (2.5ug/kg)	Sertoli cell toxicity shown by thinner epithelium and reduced germ cell layers. Decreased Anogenital distance (AGD)	Patrick et al., (169)
Paracetamol mixed with EDCs: Estrogenic (BPA, 4-MBC, butyl paraben, OMC). Anti-androgenic: DBP, DEHP, vinclozolin, prochloraz, procymidone, linuron, pp’DDE, epoxiconazole	*In utero*; Rats; oral gavage from gestation (GD7-21), and lactation (PND 1-22), using 3 doses of total mixture (TotalMix100, 200, 450). Doses in (mg/kg/day). Mix of 4 estrogens (4-MBC) (6, 12, 27), (BPA (0.15; 0.3; 0.6) butyl paraben (6, 12, 27), octyl methoxycinnamate (OMC) (12, 24, 54). 8 anti-androgens: DBP, DEHP, vinclozolin, prochloraz, procymidone, linuron, pp’DDE, epoxiconazole (3 doses each; values between 1 and 9); paracetamol (80, 160, 360)	Delayed Sertoli cell maturation in adult testes. Some rats at PND300 showed Sertoli cell only phenotype in ST after exposure to EDCs i.e. DBP, DEHP, pesticides, and paracetamol mixtures. Paracetamol decreased sperm count	Axelstad et al., (170)
Fungicide mixtures: Carbendazim, Iprodione	*Ex vivo*; Pubertal (PND20-22) rats. seminiferous tubules (ST) cultured. Exposed to CBZ and IPR (50 nM each) alone or mixed, over 3-week period.	Sertoli cell markers Cx43 and Claudin-11 decreased protein expression in exposed STs. Altered Sertoli cell and germ cell populations	Durand et al., (171)
Fungicide mixtures: Triazole, imidazole fungicides	*In vitro*; Mouse immortalized immature Sertoli TM4 cell line	Inhibition of Sertoli cell proliferation, cell cycle arrest, mitochondrial dysfunction, ROS generation, glutathione depletion, apoptosis.	Petricca et al., (172)

genistein, (GEN); di-2(ethylhexyl) phthalate, (DEHP); di(n-butyl) phthalate, (DBP); nonylphenol, (NP); mono-butyl phthalate, (MBP); 1,1,1-trichloro-2,2-bis(p-chlorophenyl) ethane. (DDT).

### 4.1 Cadmium-diazinon mixtures

The environmental metal Cadmium, to which people can be exposed *via* food, was studied alone or in combination with the organophosphate insecticide diazinon, commonly used on fruits and crops, which could also be present in food, by exposing 4-week-old rats to both chemicals in sub-chronic doses over a 90-day period *via* drinking water (161). In rats exposed to Cadmium or diazinon, germ cell sloughing, and cell necrosis were observed, and immunohistochemical analysis revealed the presence of seminiferous tubules displaying progressive damage, lacking germ cells and presenting Sertoli cell only phenotype. However, no synergy or additivity was found in rats exposed to the mixtures, which appeared to have lesser effects than the chemicals alone, suggesting that in this particular case, exposure to the mixture presented less reproductive health risk than exposure to each chemical alone.

### 4.2 Genistein and DEHP mixtures

Postnatal exposure of infants can happen in a clinical setting through soy-formula containing the phytoestrogen GEN, whilst receiving medical intervention with phthalate-containing medical tubing and equipment. DEHP which is not covalently bound, leaches onto the fluids and can reach out the infant *via* ingestion during medical intervention. Jones et al. performed *in utero* exposures studies in Sprague-Dawley rat with GEN and DEHP mixtures at 10mg/kg/day, observing morphological changes, as well as transcriptome changes and inflammatory responses in testes of offspring (162). The study identified Sertoli cells as one of the target testicular cell types, based on alteration of mRNA expression for Sertoli markers *Amh* and *Wt1*, in adult PND120 rats. Following this study, Jones et al. performed *in utero* exposure to GEN and DEHP at the same dosing as previously mentioned to further analyze the effects of GEN-DEHP mixtures in PND3 and PND6 rat testes, representing early gene responses. While various genes were altered, including antioxidant genes, it is interesting that the authors saw a decreased expression of ABP after exposure to either DEHP alone or GEN-DEHP mixture in PND6 rat testes. They also observed Wt-1 gene expression to be decreased significantly after exposure to DEHP alone but not GEN+DEHP mixture in PND3 rat testes. To complement these studies, Walker et al. analyzed the testes of adult rats exposed *in utero* to 0.1 and 10 mg/kg/day GEN+DEHP mixtures and observed an increased rate of abnormal testicular phenotypes characterized by Sertoli cells only in EDC-treated rats (164).

### 4.3 DBP and Nonyl Phenol mixtures

Another type of phthalate, DBP (and its metabolite MBP), was studied in mixture with nonyl phenol (NP) which has weak estrogenic activity, on Sertoli cells isolated from prepubertal rats (165). While they reported no remarkable changes on Sertoli cell morphology, a decreased cell viability was observed by Cell Counting Kit-8 (CCK-8) assay after 24-hour exposure to high concentrations of DBP (100 µM) and NP (10 µM) mixture. Additionally, the effects of NP and MBP were studied *in vitro* by treating 30-day-old primary rat Sertoli cells and analyzing the data *via* two mathematical models, Loewe additivity (LA) and Bliss Independence (BI) theory, used during risk assessments of EDCs (166). The authors concluded that these two mathematical models were able to indicate whether the combined effect of NP+DBP mixture had an antagonistic effect on cell viability and lactate dehydrogenase (LDH) leakage assay, which evaluates plasma membrane integrity. Furthermore, the authors extended their previous study of NP+MBP mixtures by performing an *in vitro* analysis of primary rat Sertoli cells simultaneously with an *in vivo* analysis of PND23-35 rats exposed to the same chemicals, using the BI model to quantify their data (167). The comparison of *in vitro* and *in vivo* Sertoli cell dysregulation by NP+MBP mixtures suggested that the BI model was able to predict interactions between estrogenic and antiandrogenic effects of the chemicals.

### 4.4 Phthalate mixtures

Kumar et al. performed a study to determine the effects of exposure to a mixture of three phthalates: diethyl phthalate (DEP), diphenyl phthalate (DPP), and dimethyl isophthalate (DMIP), using both *in vivo* and *in vitro* treatment of immature Sertoli cells (168). Data collected from *in vitro* treatment of 3-week-old primary mouse Sertoli cells exposed for 24 hours to the phthalate mixture showed some similar trends to the *in vivo* data on mRNA expression for various Sertoli cell markers. Additionally, *in vitro* exposure to the phthalate mixture was suggested to alter the structural and functional integrity of primary Sertoli cell culture, as seen in transmission electron microscopy (TEM) data.

### 4.5 Paracetamol and EDCs

Paracetamol/acetaminophen is an over-the-counter analgesic and antipyretic drug widely used by pregnant women and young children, that has been reported in epidemiological studies to correlate with an increased incidence of male reproductive disorders. Since these disorders are also associated with exposure to EDCs, Axelastad et al. examined the effects of *in utero* exposure to paracetamol alone and in combination with estrogenic and antiandrogenic EDCs, to explore the effects of these mixtures on male fertility (170). Mostly concerned with changes in sperm count post-exposure to mixtures, the authors found that the testes of rats exposed to estrogens, antiandrogens, and paracetamol mixtures presented seminiferous tubules with reduced germinal epithelium thickness and Sertoli-cell only phenotypes. Individual pesticides or insecticides are known to affect male reproduction, which can be further impacted by exposure to mixtures of pesticides. Patrick et al. used mixtures mimicking the exposure conditions of field workers in South Africa, where malaria as a public health threat (169). These areas have significant EDCs contaminants including the anti-malaria insecticides 1,1,1-trichloro-2,2-bis(p-chlorophenyl) ethane (DDT), 1,1-dichloro-2,2-bis(p-chlorophenyl)ethylene (DDE), and deltamethrin (DM). Additionally, people living in these areas are highly exposed to nonylphenol and cultural diets containing coumestrol, genistein, and zearalenone. Based on *in utero*, lactational, and direct exposure to these EDCs as mixtures, seminiferous tubules were found to have larger luminal sizes, suggesting Sertoli cell toxicity and altered fluid retention. In comparison, Durand et al. (2017) studied the mixed effects of two common fungicides carbendazim (CBZ) and iprodione (IPR), as androgenic and anti-androgenic prototypes, on pubertal rat seminiferous tubules (STs) in culture (171). E*x vivo* treatment of cultured STs from prepubertal rats that were exposed to CBZ, IPR, or the mixture, showed altered ratios of germ to somatic cell (Sertoli) populations and reduced levels of key junctional proteins Cx43 and Claudin 11 in samples exposed to the mixture. Lastly, Petricca et al. reported the dysregulation of TM4 Sertoli cell proliferation, cell cycle arrest, and mitochondria dysfunction in response to treatments with fungicides triazoles, ketoconazole and miconazole and imidazole, prochloraz as mixtures, showing synergistic effects (172).

## 4 Discussion and concluding remarks

**Figure 1:**
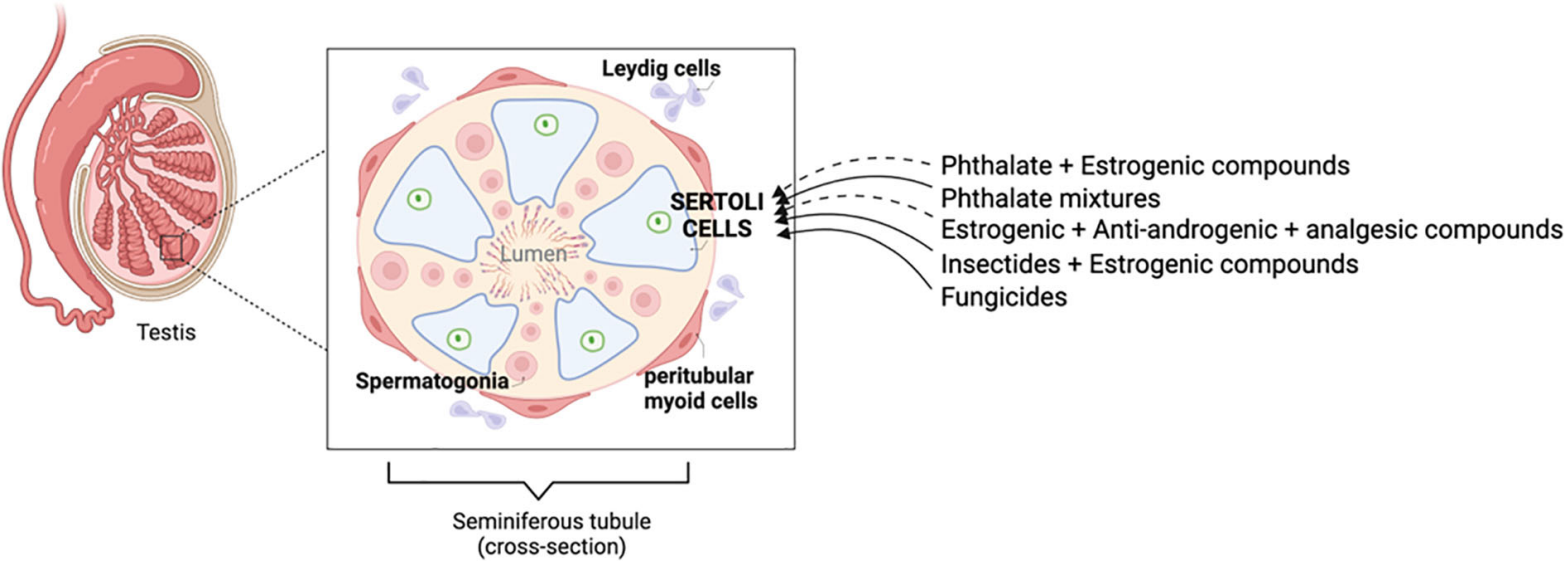
Toxicant mixtures targeting Sertoli cells in testis. The zoom-in illustrates various testicular cell types, including Leydig and peritubular myoid cells in the interstitium, and in the seminiferous tubules, Sertoli cells and germ cells, from spermatogonia to subsequent spermatogenic cells resulting in spermatozoa, which are released in the lumen. Sertoli cells play a vital role during male gonad development and throughout spermatogenesis in adulthood, acting as “nurse cells” of germ cells. Thus, assessing the impact of EDC and drug exposures on these cells is important. Some of the mixtures discussed in this review are highlighted here. A dashed arrow suggests indirect effects of exposure to toxicant mixtures on Sertoli cells, whereas a solid arrow suggests direct effects of exposure to toxicant mixtures on Sertoli cells.

This review summarizes the mechanisms regulating Sertoli cell development and functions from fetal to postnatal ages, emphasizing their pivotal role in regulating and sustaining spermatogenesis, and provides a literature survey on the adverse effects of fetal and perinatal exposure to EDCs and drugs on Sertoli cells. Although studies on individual chemicals can be found relatively easily, the same cannot be said on the effects of EDC mixtures and more complex paradigms mixing EDCs and drugs, on Sertoli cells at different ages. The fact that humans are commonly exposed to such mixtures warrants more research to close these gaps of knowledge. Several studies looking at EDC mixtures and EDC-drugs mixtures are discussed here (**Figure 1**). Additive and synergistic effects of some of these mixtures have been reported, further underscoring the need for performing risk assessment with mixtures, in addition to individual chemicals. Although

While studies have clearly demonstrated that EDCs target Sertoli cells in animal models, there is a lack of epidemiological and demographic data examining the contribution of Sertoli cells to adverse phenotypes due to EDC exposure in human. However, correlations can be inferred from studies reporting adverse effects of EDCs on genes and functional pathways known to play a role in Sertoli cell, and epidemiological data associating the disruption of these genes and pathways with male infertility in human. For example, while the negative feedback of Sertoli cell-produced Inhibin B on the hypothalamus and pituitary and its regulatory role on testicular functions is well-established, a study reported the predictive value of circulating Inhibin B levels for male infertility in human, based on the finding that infertile men had lower levels of systemic Inhibin B than fertile men (173).

Other studies have questioned the possible role of xenobiotic exposure in humans by examining the expression levels of nuclear receptors known to bind EDCs in parallel to assessing male fertility. This is the case of a study that compared the expression levels of the Aryl hydrocarbon receptor (AhR) in fertile men and men with unexplained infertility (174). AhR binds EDCs such as tetrachlorodibenzo-p-dioxin (TCDD) and Polycyclic Aromatic Hydrocarbons (PAHs), known to target Sertoli and Leydig cells and to increase male infertility in animal models. The authors reported increased levels on AhR in Sertoli, Leydig and spermatid cells in testicular biopsies of infertile men in comparison to fertile men, proposing the measurement of AhR expression as a diagnostic biomarker for idiopathic male infertility. These studies illustrate the convergence of animal studies identifying hormones and nuclear receptors targeted by EDCs in Sertoli cells with male infertility in human, in which the altered status of the same proteins correlates with infertility, suggesting EDC exposures as possible causes of the diseases.

In recent years, the idea of combining the identification of new diagnostic tools with the development of new therapies has emerged, defined as “theragnostics”, principally applied to pharmaceutical research, where a theragnostic target can be a single molecule or its interactome that can be used both as diagnostic and therapeutic tool, applied to a specific disease, and devoid of human toxicity (175, 176). An example of that is the identification of NOX5-induced uncoupling of endothelial NO synthase identified both as a cause and potential therapeutic target of age-related hypertension (177). Recently, the usefulness of the theragnosis strategy has been extended to the determination of toxicant targets related to disease, that could be used both in the prediction/diagnosis of toxicant-associated disease and as therapeutic targets countering the toxicant adverse effects. This was convincingly illustrated in a study where Keratin-18 level in serum was identified as a diagnostic and prognostic of alcohol-associated hepatitis and further proposed as theragnosis marker for predicting the efficacy of prednisolone therapy (178).

Applying the same concept to Sertoli cell toxicity in relation to male infertility could be advantageous. Indeed, using the example above of lower Inhibin B blood levels correlating with male infertility in human, and findings that inhibin B was reduced in rodents exposed to EDCs suggest that Inhibin B could potentially constitute a theragnostic, providing both a diagnostic helping to recognize cases of infertility involving Sertoli cell disruption, as well as a possible therapeutic tool. For example, since low circulating inhibin B levels correlated with abnormal spermatogenesis and infertility, identifying low Inhibin B levels could warn of possible adverse effects of a test compound on Sertoli cells, and one could aim at increasing circulating Inhibin B by upregulating its production by Sertoli cells. However, such intervention would require a clear understanding of the mechanisms regulating the production of both alpha and beta B subunits of Inhibin B, and the formation of the functional alpha-beta B dimer. Then, on could target the molecular pathway(s) regulating these processes. On the other end, in case when Inhibin B is abnormally elevated such as in inflammatory conditions (179), which have been associated with EDC exposures, one could try reducing the excess circulating Inhibin B by immunoneutralization with a specific anti-inhibin B monoclonal antibody (targeting either the alpha subunit or the dimer), or by modulating its regulation, as a way to reduce the negative feedback of Inhibin B on the anterior pituitary that blocks FSH production.

However, the production of adult Inhibin B is differently regulated than in younger ages from birth to puberty: while testosterone and germ cells contribute to Inhibin B regulation in adult, FSH plays a major role in infancy to childhood, where a positive relationship is observed between Inhibin B and FSH, in contrast to the negative feedback typical of adulthood (180). As highlighted in this review, the effects of EDCs in perinatal periods and adulthood on Sertoli cells are very different, with the ability of immature Sertoli cells to proliferate or differentiate being primary targets at young ages, whereas a primary target in the adult Sertoli cells is the maintenance of a functional BTB. Thus, identifying specific functional pathways and proteins that are disrupted as part of the mechanisms of toxicity of EDCs or pharmaceuticals in immature vs differentiated adult Sertoli cells is essential.

The combination of *in vitro* and *ex vivo* Sertoli cell models corresponding to critical developmental periods, in association with transcriptomics, epigenetics, proteomics and metabolomic approaches, computer modeling and system biology, should help understanding the mechanisms driving harmful EDC and mixed toxicants effects on the male reproductive system, and performing more powerful risk assessment (181).

## Author contributions

MC-H and MC contributed to the design and concept of the review. MC-H performed most of the literature search, wrote the first draft of the manuscript and prepared the table and figure. MC participated to the literature search and edited the manuscript and the table. All authors contributed to the article and approved the submitted version.
